# Your Patient Has a New Health App? Start With Its Data Source

**DOI:** 10.2196/14288

**Published:** 2019-06-17

**Authors:** Keith E Morse, Jonathan Schremp, Natalie M Pageler, Jonathan P Palma

**Affiliations:** 1 Department of Pediatrics Stanford University School of Medicine Palo Alto, CA United States; 2 Department of Clinical Informatics Lucile Packard Children’s Hospital Palo Alto, CA United States; 3 Division of Pediatric Critical Care Medicine Department of Pediatrics Stanford University School of Medicine Palo Alto, CA United States; 4 Division of Neonatal and Developmental Medicine Department of Pediatrics Stanford University School of Medicine Palo Alto, CA United States

**Keywords:** electronic health records, clinical informatics, health information technology

## Abstract

Recent regulatory and technological advances have enabled a new era of health apps that are controlled by patients and contain valuable health information. These health apps will be numerous and use novel interfaces that appeal to patients but will likely be unfamiliar to practitioners. We posit that understanding the origin of the health data is the most meaningful and versatile way for physicians to understand and effectively use these apps in patient care. This will allow providers to better support patients and encourage patient engagement in their own care.

With the orange ostrich only a step behind, the purple bear crosses the finish line and wins the first prize. The 9-year-old patient looks up from her smartphone and celebrates by giving her father a high-five. The father explains to her doctor that the game is an app recommended by her weight management clinic and winning the race indicates that she met her health management goals for the previous week. Her avatar, the purple bear, gets faster when she logs her daily activities, including exercise and healthy meals. Following the weight management clinic visits, the girl’s weight, blood pressure, and screening laboratory results are downloaded into the app, and the purple bear gets faster when certain goals are met. A *Summary* screen in the app allows the doctor to quickly review the patient’s health data.

This hypothetical app illustrates 2 features of a new generation of health apps: First, the outward appearance and functionality are novel. As health app development continues to gain momentum, creative, new apps will more routinely make their way into patient devices and the clinic. Second, the data that drive the app include both patient-entered health information and data downloaded from the clinic’s electronic health record (EHR). The availability of these data sources enables app developers to build innovative tools to visualize, monitor, and better utilize the data. It will be incumbent upon providers to acknowledge this new generation of health apps and consider how to incorporate them into clinical practice. The first step in the evaluation of a new app is to understand the source of its health data.

The ability for an app to access patient clinical data from the EHR is the end result of tremendous coordinated efforts between the Centers for Medicare and Medicaid Services (CMS), EHR vendors, and health systems. CMS, via the Promoting Interoperability Program, are incentivizing hospitals to allow patients to view, download, and share their health information from the EHR “using any application of their choice that is configured to meet the technical specifications of the application programming interface (API)” [1]. APIs are software interfaces that allow 2 apps to communicate—in this case, sharing data between an EHR and a health app. According to the Office of the National Coordinator for Health Information Technology (ONC), 87% of hospitals nationally use EHR platforms that provide these APIs, which are built with an interoperability standard called FHIR (Fast Healthcare Interoperability Resource) [2].

Once enabled by a health care system, these FHIR (pronounced “fire”) APIs can be accessed by patient-facing health apps. With the appropriate permission from the patient, these apps can download the patient’s Common Clinical Data Set (CCDS), an ONC-defined set of health information that includes data such as patient name, problem list, vital signs, medications, laboratory results, immunizations, and allergies [3].

Quantifying the number of hospitals that have enabled FHIR API capabilities is difficult owing to the lack of a centralized registry. Perhaps the best snapshot comes from an EHR vendor that publishes a list of customers with established FHIR API connections. At present, that list contains approximately 290 organizations, suggesting the broad adoption of FHIR APIs by customers of at least 1 major EHR vendor [4]. Elsewhere, estimates suggest that just under 200 million Americans currently have the option to download their health data [5].

When presented with a health app populated by data from an EHR, providers should keep several factors in mind. First, although FHIR APIs are widely available, they are not universally implemented. If a patient is seen at a facility without functioning FHIR APIs, the data available to the app will be incomplete. Second, patients must connect the app to their health system’s FHIR API to download available health information and must continue to initiate additional downloads when new data become available (FHIR does not yet support automatic transmission of updated EHR data). Failure of any of these steps would also result in incomplete retrieval of health information. Finally, in the care of children and other settings involving care by proxy, it is important that the correct patient’s health data are linked to the app (ie, the child’s data, not the parent’s).

The second sphere of health information is data collected by the patient and entered directly into the health app. This includes information such as logs of meals eaten, headache symptoms, or menses. More sophisticated data capture is possible if an app is paired with a connected health device that logs weight, blood pressure, physical activity, or blood glucose and passively transmits data to the app. Although transferring this health information from an app into the EHR is technically feasible and is being used in select circumstances [6], the clinical workflow and medicolegal complexities (eg, the responsibility of a provider to monitor and respond to the data) of this functionality have delayed its adoption.

Health apps that rely on patient-reported data introduce similar challenges to those of taking an oral medical history in that inaccuracies, unintentional or otherwise, can limit the reliability of the information. This problem is somewhat ameliorated by apps that are digitally connected to home monitoring devices [7]. Even in this case, an error can be introduced through improper use of a connected device (eg, blood pressures obtained from an inappropriately placed blood pressure cuff).

Although health apps with racing zoo animals remain a vision of the future, patient-facing health apps that integrate EHR and patient-generated data to support disease management are here. How these apps will be incorporated into clinical care remains to be seen [8], but the widespread adoption of the FHIR standard and the continued ability of patients to collect their own data encourage novel app development and means providers will increasingly encounter patients using health apps. Physicians need to understand the source of the health information recorded in these apps to assess their relevance and validity. Then, in conjunction with patients and families, providers will be better positioned to identify how to use these apps to best support the health of their patients. 

## Abbreviations

API: application programming interface
CCDS: Common Clinical Data Set
CMS: Centers for Medicare and Medicaid Services
EHR: electronic health record
FHIR: Fast Healthcare Interoperability Resource
ONC: Office of the National Coordinator for Health Information Technology

## References

[1] (2018). Centers for Medicare & Medicaid Services.

[2] Posnack S, Barker W (2018). The Office of the National Coordinator for Health Information Technology.

[3] The Office of the National Coordinator for Health Information Technology.

[4] Epic FHIR API Endpoints.

[5] @carinalliance (2019). Twitter.

[6] Palma J, Pageler N (2018). Stanford University.

[7] Bonafide CP, Jamison DT, Foglia EE (2017). The emerging market of smartphone-integrated infant physiologic monitors. J Am Med Assoc.

[8] Dameff C, Clay B, Longhurst CA (2019). Personal health records: more promising in the smartphone era?. J Am Med Assoc.

